# Body dimensions of the extinct giant shark *Otodus megalodon*: a 2D reconstruction

**DOI:** 10.1038/s41598-020-71387-y

**Published:** 2020-09-03

**Authors:** Jack A. Cooper, Catalina Pimiento, Humberto G. Ferrón, Michael J. Benton

**Affiliations:** 1 School of Earth Sciences, University of Bristol, Bristol, BS8 1RJ UK; 2 Department of Biosciences, Swansea University, Swansea, SA28PP UK; 3 Smithsonian Tropical Research Institution, Balboa, Panama; 4 Paleontological Institute and Museum, University of Zurich, Zurich, CH-8006 Switzerland

**Keywords:** Palaeontology, Ichthyology

## Abstract

Inferring the size of extinct animals is fraught with danger, especially when they were much larger than their modern relatives. Such extrapolations are particularly risky when allometry is present. The extinct giant shark †*Otodus megalodon* is known almost exclusively from fossilised teeth. Estimates of †*O. megalodon* body size have been made from its teeth, using the great white shark (*Carcharodon carcharias*) as the only modern analogue. This can be problematic as the two species likely belong to different families, and the position of the †*Otodus* lineage within Lamniformes is unclear. Here, we infer †*O. megalodon* body dimensions based on anatomical measurements of five ecologically and physiologically similar extant lamniforms: *Carcharodon carcharias*, *Isurus oxyrinchus*, *Isurus paucus*, *Lamna ditropis* and *Lamna nasus*. We first assessed for allometry in all analogues using linear regressions and geometric morphometric analyses. Finding no evidence of allometry, we made morphological extrapolations to infer body dimensions of †*O. megalodon* at different sizes. Our results suggest that a 16 m †*O. megalodon* likely had a head ~ 4.65 m long, a dorsal fin ~ 1.62 m tall and a tail ~ 3.85 m high. Morphometric analyses further suggest that its dorsal and caudal fins were adapted for swift predatory locomotion and long-swimming periods.

## Introduction

Estimating the body size of exceptionally large extinct taxa is a difficult task because the fossil record is inherently incomplete and because allometry, if present, can make extrapolations hard to model. Palaeontologists therefore have to rely on the relationships between often isolated and fragmented body-part remains and length in extant relatives to estimate the body size of extinct giants^1, 2^. The extinct †*Otodus megalodon* has been estimated to be the largest macropredatory shark known to have existed^3^. Based on its fossil teeth and using the modern great white shark (*Carcharodon carcharias*) as an analogue, it has been calculated that it reached a maximum total length (herein, TL) of ~ 15 to 18 m^3–5^.

†*Otodus megalodon* was originally classified in the family Lamnidae (order Lamniformes) with *C. carcharias* considered its closest living relative^3, 6–8^. This classification was based on similar tooth morphologies^3, 7, 8^, which also implied that the two species shared an ecological function as apex macropredators. *Carcharodon carcharias* has therefore been widely used as the main modern analogue of †*O. megalodon*^3, 4, 9, 10^. Accordingly, linear relationships between tooth crown height and TL recorded in *C. carcharias*^5, 11^ have been used extensively to infer the size and skeletal anatomy of †*O. megalodon*^3–5, 9, 12–14^. A detailed examination of tooth morphology challenged the relationship between *C. carcharias* and †*O. megalodon*, revealing that *C. carcharias* descended from a lineage that includes the mako sharks (*Isurus* spp.) and other closely related taxa (i.e. †*Cosmopolitodus*) rather than †*O. megalodon*^15^. This hypothesis has further been supported by the fossil record of *Carcharodon*^16–19^. Accordingly, †*O. megalodon* was reassigned to the family †Otodontidae within the order Lamniformes^15, 17–25^. Given the different hypotheses for its phylogenetic placement, †*O. megalodon* has been reported in the literature under different genera such as †*Carcharocles*, †*Megaselachus* and †*Procarcharodon*^21^. We follow the hypothesis supporting the †*O. megalodon* lineage as a distinct family (†Otodontidae), derived from the extinct genus †*Cretalamna*^7, 15, 22^, and therefore use the genus †*Otodus*.

Despite the fact that the placement of †*O. megalodon* in the family †Otodontidae has been widely explored^22^, the interrelationships between otodontids and other lamniforms remain questionable^25^. This uncertainty, coupled with the fact that sharks of different sizes have been reported as being geometrically similar in body profile^26, 27^, suggests that other macropredatory lamniforms, in addition to *C. carcharias,* could serve as modern analogues of †*O. megalodon*, thus aiding the reconstruction of body dimensions (i.e. head length, dorsal fin height and width, tail height).

Here, based on a series of anatomical measurements from extant macropredatory lamniforms, we reconstruct the linear body dimensions of †*O. megalodon* at different life stages. We do this using regression analyses of body parts as a function of TL, which have been previously used for both morphological scaling of body form^26, 27^, and to predict nonlinear variables such as body mass in sharks^28^. To select our additional analogues alongside *C. carcharias*, we utilise extant phylogenetic bracketing^29^. This method allows us to base our chosen taxa on shared traits between the extant and extinct taxa—in this case, dental, ecological and physiological similarities^24, 25^. We therefore select the five extant species of family Lamnidae^30^ as our analogues based on their shared traits with †*O. megalodon* (see “Methods” for more details). Our results reveal the potential measurements of (and distances between) body parts given different total lengths (i.e., 3, 8 and 16 m). The estimates of body dimensions of this extinct species have the potential to inform future anatomical, physiological and ecological reconstructions.

## Results

### Allometry: linear regressions and morphometrics

We first tested for allometry within and between species by modelling 24 anatomical measurements (Supplementary Table S1, S2; Supplementary Data 1, 2) as functions of TL in all five modern analogues (*Carcharodon carcharias*, *Isurus oxyrinchus*, *Isurus paucus*, *Lamna ditropis* and *Lamna nasus*). Measurements in all species showed positive linear relationships with TL, with no evidence for allometry between or within species (Supplementary Fig. S1). Similar relationships were observed within individual life stages (i.e. juveniles, subadults and adults; Supplementary Figs. S2–S4). The slope of all linear regressions overlapped, ranging within ~ 0.1 units of one another between species (Supplementary Fig. S5). Adjusted R^2^ values were relatively high, with 89% of them over 0.7 and 62% over 0.9 (Supplementary Data 3). Of the 144 recorded linear relationships, only six did not show statistical significance (Supplementary Data 3). The most statistically significant linear regressions came from the model using data from all analogue species (*P* < 0.01; Supplementary Data 3).

**Figure 1:**
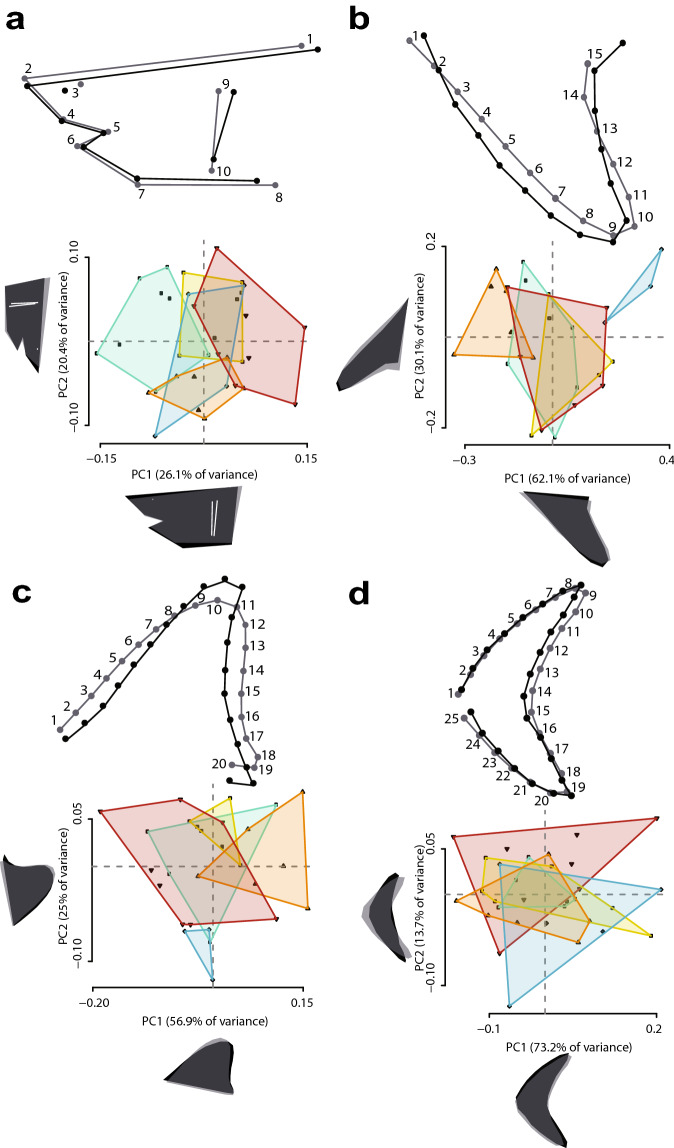
Regression shape changes (above) and PCA (below) of Procrustes coordinates for the five analogue species. These are recorded in the (**a**) head, (**b**) pectoral fin, (**c**) dorsal fin, and (**d**) caudal fin. In the regression analyses, light and dark grey configurations represent the morphological change occurring from the average shape towards higher scores, considering in all cases a magnitude of the shape change equal to 0.1. Individual colours represent each species in the PCA: green = *Carcharodon carcharias*; yellow = *Isurus oxyrinchus*; blue = *Isurus paucus*; orange = *Lamna ditropis*; red = *Lamna nasus*.

To complement the linear regressions, we used geometric morphometrics to evaluate the morphology of the head and fins of the five analogue species, and performed regression analyses between shape and TL to assess for allometry (see “Methods”). A principal component analysis (PCA) revealed shared morphospace in all body parts tested (Fig. 1), the only exception being *I. paucus* (Fig. 1b,c). Morphological variability between our species is explained by changes in the length of the snout and robustness of the head, in the span and length of the pectoral and dorsal fin, and in the relative length of the dorsal and ventral lobe and the span of the caudal fin. The regression analyses indicate that larger analogues had slightly more robust heads (Fig. 1a; *P* = 0.1106) and more convex dorsal fins (Fig. 1c; *P* = 0.0038), whereas smaller analogues had slender heads and more concave rear edges in the dorsal fins (Fig. 1a,c). No allometric change was detected in the pectoral (Fig. 1b; *P* = 0.5924) or caudal fin (Fig. 1d; *P* = 0.3208). The caudal fin was found to be the same dorsally directed shape in all analogues (Fig. 1d). All of these results were also observed when all landmarks (total body) were analysed together within a single configuration (Supplementary Fig. S6), with no allometric change detected (*P* = 0.3028).

### 2D reconstruction of linear body dimensions

**Figure 2:**
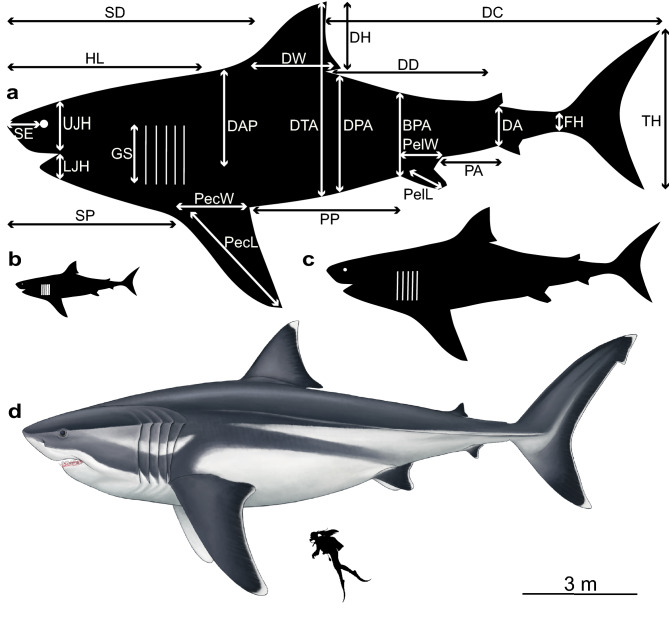
Silhouette models visualising †*Otodus megalodon* body dimensions based on our extrapolations at different total lengths. (**a**)  ~ 16 m, (**b**)  ~ 3 m and (**c**)  ~ 8 m. Abbreviations as in Table 1. Silhouettes created in Adobe Illustrator CC 2018. (**d**) Palaeoartistic reconstruction of a 16 m †*O. megalodon* scaled against a 1.65 m human (illustration by Oliver E. Demuth). Fin shapes are based on our generalised morphometric shapes in the silhouettes whereas the reconstruction aims to capture their true biological shapes, i.e. the ceratotrichia of the tail present in all five modern analogues.

The best linear model (highest statistical significance by 7–33 orders of magnitude; see above and Supplementary Data 3) came from the regression that uses all five analogues together and it is therefore the basis for our extrapolations. We visualise our extrapolations in silhouetted shark models, and in a palaeoartistic reconstruction that also considers our generalised fin and head shape changes in relation to TL uncovered in our morphometric analyses (Fig. 2). Converting the anatomical measurements of our analogues into proportions based on TL indicate that a mature, 16 m †*O. megalodon* would have had a head ~ 4.65 ± 0.42 m long (~ 29% TL), a dorsal fin ~ 1.62 ± 0.36 m tall (~ 10% TL) and 1.99 ± 0.3 m wide (~ 12% TL), a height of 4.53 ± 0.56 m (~ 28% TL) from the tip of the dorsal fin to the abdomen, and a tail ~ 3.85 ± 0.7 m high (~ 24% TL) (Fig. 2a; Table 1). These measurements for a neonate (3 m; Fig. 2b) and a juvenile †*O. megalodon* (8 m; Fig. 2c) can be found in Table 1. No dimension in individual life stages overlap within the predicted ranges of other stages (mean ± standard deviation). Our model of a 16 m †*O. megalodon* using all analogues was stockier (wider vertical dimensions; Supplementary Table S3) and had stronger statistical support (by 7–29 orders of magnitude; see Supplementary Data 3) than an alternative model based on *C. carcharias* only (the sole analogue previously used). Finally, this multi-analogue model accurately predicted 22/24 of the dimensions of a *C. carcharias* of known size (Supplementary Table S4).

**Table 1:** Proportional mean and standard deviation of all variables against TL and their extrapolations to a 3 m, 8 m and 16 m *O. megalodon*.

Variables	Mean proportion	Sd proportion	Mean (3 m)	SD (3 m)	Mean (8 m)	SD (8 m)	Mean (16 m)	SD (16 m)
HL	0.29	0.03	87.16	7.82	232.43	20.86	464.86	41.71
SE	0.05	0.02	14.94	4.79	39.85	12.77	79.69	25.55
UJH	0.07	0.01	22.25	3.26	59.33	8.7	118.66	17.39
LJH	0.04	0.01	10.68	4.08	28.47	10.89	56.95	21.78
SP	0.26	0.03	79.06	8.65	210.84	23.08	421.68	46.16
GS	0.09	0.01	26.47	4.25	70.6	11.33	141.19	22.66
PecL	0.19	0.05	57.83	14.48	154.21	38.61	308.42	77.22
PecW	0.1	0.02	30.9	5.87	82.4	15.65	164.79	31.29
SD	0.37	0.03	111.32	9.4	296.85	25.08	593.71	50.15
DH	0.1	0.02	30.47	6.66	81.24	17.77	162.48	35.53
DW	0.12	0.02	37.31	5.63	99.48	15.01	198.96	30.01
DAP	0.15	0.02	43.6	6.39	116.27	17.04	232.54	34.09
DTA	0.28	0.03	84.96	10.45	226.55	27.88	453.11	55.76
DPA	0.18	0.03	52.58	7.55	140.21	20.13	280.41	40.26
DD	0.23	0.02	70.02	6.22	186.71	16.6	373.43	33.19
PP	0.22	0.04	65.98	10.92	175.96	29.12	351.92	58.24
PelL	0.05	0.01	13.79	3.41	36.78	9.11	73.56	18.21
PelW	0.06	0.02	18.71	4.58	49.89	12.22	99.77	24.45
BPA	0.12	0.01	36.74	3.66	97.99	9.75	195.97	19.5
PA	0.09	0.02	28.19	5.25	75.18	14.01	150.35	28.01
DA	0.06	0.01	17.73	1.8	47.29	4.81	94.58	9.62
DC	0.51	0.03	151.58	8.82	404.22	23.52	808.43	47.04
FH	0.03	0.003	8.19	0.91	21.84	2.43	43.69	4.86
TH	0.24	0.04	72.26	13.08	192.68	34.89	385.36	69.78

Measurements are in cm and accurate to two decimal places.

*HL* head length, *SE* snout-eye distance, *UJH* upper jaw height, *LJH* lower jaw height, *SP* snout-pectoral fin distance, *GS* gill size, *PecL* pectoral fin length, *PecW* pectoral fin width, *SD* snout-dorsal fin distance, *DH* dorsal fin height, *DW* dorsal fin width, *DAP* dorsal anterior-pectoral fin distance, *DTA* dorsal tip-abdomen distance, *DPA* dorsal posterior-abdomen distance, *DD* primary-secondary dorsal fin distance, *PP* pectoral-pelvic fin distance, *PelL* pelvic fin length, *PelW* pelvic fin width, *BPA* dorsal side-pelvic fin anterior distance, *PA* pelvic-anal fin distance, *DA* secondary dorsal-anal fin distance, *DC* dorsal-caudal fin distance, *FH* fork height, *TH* tail height.

## Discussion

The lack of allometry in morphological measurements and strong correlations between the measured variables and TL indicate sufficient predictability and removes risk in the use of extrapolations to estimate the body dimensions of †*O. megalodon.* The small range in the slope of all linear models implies analogous anatomical relationships between species. These results are supported by earlier suggestions that lamniform morphology strongly links to ecology^31, 32^. Therefore, our analogues share a basic external anatomy template that can be applied to †*O. megalodon*. Our extrapolations to †*O. megalodon* were based on a linear model that includes all five modern analogues. Although this may partially be due to the wider range of measurements resulting from combining the five analogues, this model statistically outperforms all others, including a model that considers *C. carcharias* only (Supplementary Table S3). Importantly, our model was proven to predict with accuracy the dimensions of a shark of known size (Supplementary Table S4).

As expected, given the presence of isometry, the dimensions of †*O. megalodon* body parts increase with TL and therefore, growth. Our calculated sizes can therefore be used to assist ecological inferences of †*O. megalodon*. It is worth noting that the largest estimated TL of †*O. megalodon* is more than twice the size of the largest living lamnid^3–5^. As such, it can be risky to use extrapolations instead of interpolations. The presence of larger living analogues (> 7 m TL) would make such extrapolations less risky, but such macropredatory lamniforms do not exist in today’s oceans^30^. However, the lack of significant allometry found in our analogues from both linear regression and geometric morphometric analyses justifies the use of extrapolations and therefore our ecological interpretations.

Morphometric analyses, albeit mainly used to aid our assessment of allometry, also revealed the possible shapes of the fins and the head in relation to TL. Two distinct dorsal fin shapes were found, with larger sharks possessing taller but narrower convex dorsal fins than smaller sharks (Fig. 1c). Convex dorsal fins in large sharks allow long cruising periods and quick bursts of speed to ambush prey^33, 34^. The enormous †*O. megalodon* therefore likely had a convex dorsal fin built for stabilising swift predatory locomotion and long-swimming periods. This kind of locomotion could have been enhanced by mesothermy, enabling sudden acceleration in predation^23–25, 34^. However, such a large shark was unlikely to have been capable of long periods of fast swimming^34^. Research in other giant extinct marine taxa such as ichthyosaurs has suggested that steadier swimming can be used by large predators to reduce energy expended in locomotion^35^. Therefore, †*O. megalodon* may have also used scavenging as a feeding strategy, especially as it grew older. Opportunistic scavenging on large whale carcasses has been recorded in *C. carcharias*, with one study^36^ noting from four occurrences over ten years that these carcasses quickly attracted large adult individuals. Based on the fact that all analogues share the same dorsally directed caudal fin shape, the same morphology was likely displayed by †*O. megalodon* (Fig. 2d). This tail anatomy has been categorised as a “type 4” among extant lamniforms^32^. Tail morphology and evolution have been proposed to be strongly linked to ecology^32^. Taken together, morphometric analyses of the fins suggest that the giant †*O. megalodon* was likely a thunniform swimmer, where swimming motion is confined to the tail for high speeds and long distance swimming^30, 32^.

In terms of the head, the distinct morphology of larger analogues suggests that the head of †*O. megalodon* was likely robust, corroborating a large-prey preference as previously proposed based on the fossil record^8, 37^. Nevertheless, given that during ontogeny †*O. megalodon* likely shifted its dietary preference from fishes to marine mammals^9, 38^, such a robust head might have particularly benefited adult individuals with high energetic demands^39^. †*Otodus megalodon*’s head would have therefore needed large muscles to support its massive jaws, likely resulting in a more curved snout than in *C. carcharias* since the body would not have been able to taper to the nose so sharply^3^. This agrees with the previous suggestion that †*O. megalodon* had a much greater bite force than that of *C. carcharias*, and perhaps the greatest bite force of any marine predator known throughout geological time^40^. Finally, based on the external colouring of extant macropredatory sharks^41^, we propose that †*O. megalodon* was likely countershaded. This would have allowed the shark to camouflage against light flow^41, 42^, hence facilitating ambush predation^8, 37, 39^ and the evasion of predators by nursery-dwelling juveniles^9, 14^. Our palaeoartistic reconstruction based on our results and ecological inferences allowed us to visualise this hypothesis, as well as the generalised fin and head shapes not captured by the silhouetted shark models (Fig. 2).

This study marks the first quantitative estimate of †*O. megalodon* specific body-part dimensions, beyond its overall body size. Our model based on a selection of modern analogues outperforms those using individual species (e.g. *C. carcharias*) and accounts for variability around body dimension averages. Our results reveal that body dimensions of our analogues isometrically correlate to TL. This finding agrees with previous discoveries of similar relationships in linear body dimensions of several other extant shark species^26, 27^. Although the exact phylogenetic relatedness of †*O. megalodon* and its family to the order Lamniformes remains poorly understood^21, 22, 25^, our chosen analogue taxa are the most ecologically and physiologically similar living species to †*O. megalodon*. As such, our ecological inferences for †*O. megalodon* are similar to those of our analogues, but also line up with what has been inferred from its fossil record^3, 8, 9, 23–25, 37, 39^. The knowledge of specific body dimensions beyond TL will therefore enhance further anatomical and ecological reconstructions of this giant marine apex-predator.

## Methods

### Analogue species

Order Lamniformes comprises 15 extant species^30^. Of these, five—the great white shark (*Carcharodon carcharias*), the shortfin mako shark (*Isurus oxyrinchus*), the longfin mako shark (*Isurus paucus*), the salmon shark (*Lamna ditropis*) and the porbeagle shark (*Lamna nasus*)—were selected as analogues to †*O. megalodon* based on dental, physiological and ecological similarities. Our analogues comprise the family Lamnidae; a group of large, fast-swimming, mesothermic, macropredatory sharks^30^. Both lamnids and otodontids, the ‘megatoothed’ lineage which †*O. megalodon* belongs to, are believed to have evolved from the family Cretoxyrhinidae ^7, 8, 21^. This family has been interpreted as mesothermic based on sea surface palaeotemperature, swim speed estimates and metabolic inferences^24^. These same methods were used for otodontids and the results suggested similar thermoregulatory capabilities^24^. Moreover, a phylogenetic analysis of the evolution of thermophysiology in this group found that mesothermy had likely evolved once in the Cretaceous^25^. Based on these studies, we considered otodontids to be mesothermic. Furthermore, the five chosen analogue taxa possess tooth morphologies similar to various otodontids, suggesting similar diet and ecology. For example, both families show variation in occurrences of dental lateral cusplets^30, 43^. Based on these variations, *L. nasus* is considered the best dental analogue for both †*Cretalamna* and †*Megalolamna*, mako sharks (*Isurus* spp.) have similar dental morphology to †*Otodus*, and *C. carcharias* has similar dentition to †*Otodus* (*Carcharocles*) and †*Otodus* (*Megaselachus*)^21^. As such, the five chosen analogues for this study share a unique physiological adaptation, ecology and dental morphology with †*O. megalodon* and other members of its proposed family.

### Data collection

We searched for images of all analogues in the Web using the species and common names. Most of these images were retrieved from online databases^44–46^. In total, we collected 54 images. The source of each image and more details can be found in Supplementary Data 1. We took 25 anatomical measurements of all individuals from digital images (Supplementary Data 2; Supplementary Table S1). Scaled image measurements of both traditional and geometric morphometrics have been previously used to respectively acquire linear body dimensions and to infer variation in morphology and ecology in marine organisms^47–50^. This method therefore represents a viable non-lethal alternative for collecting measurement data, which have been proposed as urgently needed for the declining populations of large predatory sharks^51^. The life stage of each individual was also recorded (see Supplementary information for more details). We selected the best images for our analyses using a scoring system, in which images with no distortion or blur had the highest score and from which TL was known or could be estimated using a scale. Angled specimens were tilted to a purely lateral view using ImageMagick^52^. Measurements were taken using ImageJ^53^. In total, 41 shark individuals were used (*C. carcharias*: *n* = 9; *I. oxyrinchus*: *n* = 9; *I. paucus*: *n* = 5; *L. ditropis*: *n* = 9; *L. nasus*: *n* = 9) (image score = 3; Supplementary Data 1).

### Linear regressions

We tested for allometry across all data and in individual life stages by modelling all anatomical measurements as a function of TL in R^54^. Because linear models assume normal distribution, raw data were Tukey transformed in the *rcompanion* package^55^ (see Supplementary Table S2). We retrieved the parameters of the relationship, extracting the linear regression from the model as:

$$y \, = \, mx \, + \, c$$

where x = TL, y = body measurement, m = slope and c = intercept (see Supplementary Data 3).

### Geometric morphometrics

Our geometric morphometrics approach followed similar methodology to Ferrón et al.^56^, which used allometric regression analyses of shark palaeoecological data to infer the caudal fin morphology of †*Dunkleosteus terrelli*. We defined a series of landmarks of type 1, 2 and 3 (head: *N* = 10; pectoral fin: *N* = 15; dorsal fin: *N* = 20; caudal fin: *N* = 25; total body: *N* = 68) that were digitised using tpsDig2 software^57^ (Supplementary Fig. S7). All subsequent analyses were conducted in MorphoJ^58^. The superimposition of landmark configurations was carried out with full Generalised Procrustes Analysis (GPA) and Procrustes coordinates were subjected to principal component analysis (PCA) to determine morphospace occupation shared by the analogues. The significance of the regressions was checked by means of permutation tests (*N* = 10,000). Finally, the Pinocchio effect (where variation is extremely localised to a single landmark, or a small number, and is then smeared over a wider area during least-square Procrustes superimposition^59, 60^), was checked by comparing full GPA and Resistant Fit Theta-Rho Analysis (RFTRA) superimpositions in IMP CoordGen8 software^61^. This risk of distortion was excluded by the results of these comparisons (Supplementary Fig. S8). All outlier sharks with fin abnormalities were removed from the analysis. These included images in which the pectoral fin was not in position for horizontal swimming, and, in one case, an image displaying “Lucy”, a ~ 5 m *C. carcharias* with a damaged caudal fin (Supplementary Data 2). If included, these specimens would have resulted in fin landmarks in differing positions in relative morphospace, something that can result in landmark distortion and potentially the Pinocchio effect. In all statistical analyses, we considered *P* < 0.05 as the threshold of statistical significance.

### Morphological extrapolations

We converted anatomical measurements of the five analogues to proportions based on TL. We then calculated the mean, standard deviation, maximum and minimum values of each measurement in centimetres (cm) and extrapolated them to †*O. megalodon* measuring 3 m (neonate), 8 m (juvenile)^3, 9^ and 16 m (conservative maximum body size^5^) using the linear regression described above. Sizes chosen to represent each life stage were based on ontogenetic inferences made by Gottfried et al.^3^ in their skeletal reconstruction of †*O. megalodon*. We compared our extrapolations of a 16 m long †*O. megalodon* against an alternative model that considered only *C. carcharias*. Our model’s accuracy was tested by using it to infer the body dimensions of a ~ 7 m long *C. carcharias.* Finally, we created basic silhouette models to illustrate and scale our extrapolations at each life stage, and had a palaeoartistic reconstruction made to illustrate our results and ecological inferences in a biological light (Fig. 2d).

## Supplementary information

Supplementary information

## Data Availability

The datasets generated and/or analysed during the current study can be found via the Dryad Digital Repository at: https://datadryad.org/stash/share/cGI08m4rPYWUD6VucWxu0oz3TniVnLKC-5umhvLHgaE.
