# Supplementary material for: Body dimensions of the extinct giant shark Otodus megalodon: a 2D reconstruction
Source: Sci Rep. 2020 Sep 3;10:14596. doi: 10.1038/s41598-020-71387-y (PMC7471939; doi:10.1038/s41598-020-71387-y)
Supplement: Supplementary file 1 — Supplementary information [file 41598_2020_71387_MOESM1_ESM.docx]

**Supplementary information**

**Body dimensions of the extinct giant shark *Otodus megalodon*: A 2D reconstruction**

Jack A. Cooper, Catalina Pimiento, Humberto G. Ferrón, Michael J. Benton

**This file includes:**

Supplementary Materials and Methods

Supplementary Table 1. Mean (± SE) anatomical measurements of all anatomical variables in each analogue species.

Supplementary Table 2. Normality tests of the study dataset.

Supplementary Table 3. Comparison of our model using all analogues *vs.* a model using the Great White Shark only.

Supplementary Table 4. Model accuracy test.

Supplementary Figure 1. Linear relationship between total length and all 24 anatomical variables.

Supplementary Figure 2. Linear relationship between total length and all 24 anatomical variables in juvenile analogues.

Supplementary Figure 3. Linear relationship between total length and all 24 anatomical variables in subadult analogues.

Supplementary Figure 4. Linear relationship between total length and all 24 anatomical variables in adult analogues.

Supplementary Figure 5. Slopes of linear relationship between total length and body dimensions for all species.

Supplementary Figure 6. Generalised regression shape changes and PCA for total body analysis.

Supplementary Figure 7. Landmark configurations for morphometric analyses in the head, pectoral fin, dorsal fin and caudal fin.

Supplementary Figure 8. GPA and RFTRA superimpositions for the head, pectoral fin, dorsal fin and caudal fin.

Supplementary References

**Other materials (in Dryad Repository) include:**

Supplementary Data 1. Dataset for our analogue species, measurements, image scoring, life stage (J = juvenile; S = subadult; A = adult), sex (M = male; F = female; U = uncertain) and sources for all 54 images used in our analyses.

Supplementary Data 2. Image dataset of all images measured within Supplementary Data 1.

Supplementary Data 3. Linear regressions of each anatomical variable against total length. Regressions (*N* = 144) are split into six separate models – one that contains all data and thus all five analogue species; and individual models for each analogue species. Also included are the adjusted R^2^ and p values, and extrapolations of each equation to a 16 m †*O. megalodon* where x = 1,600 cm. The six regressions that did not exhibit statistical significance (*P* > 0.05) are displayed in bold. While statistical significance is seen in 138 linear relationships, the model containing all five species is the most significant by 7-33 orders of magnitude than any individual species model in all 24 variables. Moreover, adjusted R is > 0.9 in 17/24 variables; indicating more reliable predictors of TL than any model concerning any individual species.

Dryad Digital Repository materials can be found at: [https://datadryad.org/stash/share/cGI08m4rPYWUD6VucWxu0oz3TniVnLKC-5umhvLHgaE](https://urldefense.proofpoint.com/v2/url?u=https-3A__datadryad.org_stash_share_cGI08m4rPYWUD6VucWxu0oz3TniVnLKC-2D5umhvLHgaE&d=DwMCaQ&c=sJ6xIWYx-zLMB3EPkvcnVg&r=j5rzw3I5ZrkvSOJgw1ecGCbp7BqlxpCZmRjd0zKgQ3c&m=mppfWknl17UsELQR2VpEL1qeLjfKZEuBXioOPxzHvH4&s=9MIVJDghBR9C6jTwvU7I0NHEpcpoSPHuDSnf7FyFnbU&e=)

**Supplementary Materials and Methods**

**Data collection – life stages**

Life stages were recorded within each individual shark to allow assessment for allometry within each stage. The life stage was assessed based on two factors: (1) if the life stage was stated within the source the image came from, and (2) known relationships between body size and life stage from the literature^1-5^. As such, ranges in total length (herein, TL) were determined from these two factors to classify life stage. For the great white shark (*C. carcharias*), 120-250 cm was considered juvenile; 250-350 cm was considered subadult and individuals ≥ 350 cm were classed as adults. In the shortfin mako shark (*I. oxyrinchus*), 75-150 cm was considered juvenile, 150-220 cm was classed as subadults and ≥ 220 was considered an adult. In the longfin mako shark (*I. paucus*), of which little reproductive and ontogenetic information is known^1^, 110-150 cm was classed as juvenile, 150-220 was considered subadult and ≥ 220 cm was considered adult. For the salmon shark (*L. ditropis*), 30-135 cm was considered juvenile, 135-200 was considered subadult and ≥ 200 cm was classed as adult. Finally, in the porbeagle shark (*L. nasus*), 80-140 cm was classed as juvenile, 140-190 was considered subadult and ≥ 190 cm was classed as adult.

**Supplementary Table 1. Mean anatomical measurements (± SE) of all anatomical variables in each analogue species.**

Measurements are accurate to one decimal place (cm). Parenthesis = ranges.

|  | Great White shark | Shortfin Mako shark | Longfin Mako shark | Salmon shark | Porbeagle shark |
| --- | --- | --- | --- | --- | --- |
| **TL** | 294.7 ± 44.3 (120-488.7) | 118.4 ± 13.5 (75-200) | 156.8 ± 21.1 (118.2-226.9) | 113.4 ± 21.3 (33.5-250) | 150.4 ± 15 (81-196) |
| **HL** | 85.3 ± 12.6 (32.7-132.9) | 34.6 ± 3.7 (23.7-57.3) | 42.2 ± 4.2 (33.8-54.3) | 30.5 ± 4.9 (9.8-58.2) | 46.2 ± 4.8 (25.9-72.2) |
| **SE** | 12.6 ± 2.4 (3.4-25.5) | 7.1 ± 0.9 (4.6-13.4) | 6.3 ± 0.4 (5.6-7.7) | 4.1 ± 0.6 (1.5-6.4) | 9.5 ± 1.4 (5.9-19) |
| **UJH** | 22.5 ± 3.7 (28.5-125.4) | 8.3 ± 1 (4.2-13.1) | 10.1 ± 1.5 (7-14.4) | 8.3 ± 1.3 (3-15) | 11.9 ± 1.2 (7-17.3) |
| **LJH** | 10.8 ± 2.5 (2.2-26.9) | 4 ± 0.7 (1.8-8) | 4.5 ± 0.5 (2.8-5.3) | 3.7 ± 0.8 (0.8-8.6) | 6.4 ± 0.9 (3-11) |
| **SP** | 76.3 ± 11.4 (28.5-125.4) | 30.6 ± 3.1 (20.7-49.5) | 38 ± 2.7 (32-44.8) | 28.2 ± 4.5 (9-53.1) | 42.2 ± 4.4 (24.7-68.4) |
| **GS** | 28.8 ± 4.4 (11.4-38.7) | 10 ± 1.1 (6.3-15.9) | 13.2 ± 2 (8.5-19.7) | 9.6 ± 1.9 (3.1-22.9) | 13.1 ± 1.7 (7.7-22.8) |
| **PecL** | 54.5 ± 7.3 (27-86.3) | 17.3 ± 1.7 (11.5-24.9) | 44.9 ± 6.6 (29.5-64.4) | 19.3 ± 3.2 (6.4-34.4) | 30.4 ± 3.7 (14.9-49.3) |
| **PecW** | 34.8 ± 4.8 (15.4-49) | 10.8 ± 1.4 (6.8-19.9) | 15.4 ± 3.9 (6.9-29) | 11.9 ± 2 (4-22.3) | 15.2 ± 2.3 (6.8-25.4) |
| **SD** | 113.3 ± 17 (42.3-174.8) | 45.9 ± 5 (31.6-77.7) | 60.4 ± 7.5 (46.9-84.1) | 37.5 ± 6.1 (11.9-72) | 54.4 ± 5.5 (29.3-78.8) |
| **DH** | 30.5 ± 4.8 (10.6-52.5) | 9.6 ± 1.2 (6-16.3) | 15.3 ± 2.3 (10.8-22.3) | 12.5 ± 3 (2.4-30.6) | 19.2 ± 2.6 (8.3-29.4) |
| **DW** | 36.8 ± 5.3 (16.3-58) | 12.6 ± 1.3 (8-20.2) | 16.7 ± 2.6 (12.1-23.4) | 15.2 ± 3 (4.4-32.6) | 21.7 ± 2.8 (10.7-33.8) |
| **DAP** | 42.6 ± 6.9 (16.3-73) | 16.7 ± 2.3 (8.8-27.9) | 19.1 ± 3.3 (12.5-28.5) | 17.5 ± 2.8 (5.4-33) | 23.1 ± 2.2 (13.1-30.6) |
| **DTA** | 80.2 ± 12.1 (34.3-125) | 30 ± 3.8 (18.4-50.4) | 43.1 ± 7.5 (30.2-65.1) | 33.7 ± 6.8 (9.9-78) | 48.9 ± 5.2 (24.6-67.8) |
| **DPA** | 47.7 ± 7.5 (19.9-85.5) | 20.5 ± 2.8 (11.2-35.3) | 26.5 ± 4.8 (18.5-40.9) | 20.5 ± 3.9 (6-46.5) | 28.2 ± 2.5 (16.3-35.9) |
| **DD** | 60.8 ± 9 (26.9-97.4) | 29.1 ± 3.5 (18-48) | 38.7 ± 4.6 (30.2-53.1) | 28.1 ± 6 (7.9-69.4) | 35 ± 3.5 (18.2-46.6) |
| **PP** | 63 ± 11.5 (23.7-135) | 28.7 ± 3.4 (17.7-48.1) | 38.2 ± 7.1 (25.6-59) | 24.1 ± 4.8 (6.3-48.4) | 30.9 ± 2.8 (17.3-40.3) |
| **PelL** | 15.8 ± 2.5 (7.1-29.7) | 4.6 ± 0.7 (2.4-7.8) | 6.1 ± 0.6 (4.2-7.6) | 5.2 ± 1.2 (1.7-13.5) | 7.6 ± 1.1 (1.5-10.8) |
| **PelW** | 19.7 ± 2.5 (10.7-31.3) | 6.6 ± 1.3 (1.9-13.9) | 9.5 ± 1.1 (7.6-13.5) | 7.5 ± 1.5 (2.2-17.1) | 9.6 ± 1.4 (2.7-14.6) |
| **BPA** | 33.3 ± 4.9 (13.7-51.5) | 14.7 ± 1.8 (8.9-26.3) | 19.8 ± 3.1 (13.7-29.2) | 14.3 ± 2.8 (4-32.8) | 19 ± 2 (9.9-25.3) |
| **PA** | 25.8 ± 3.9 (10.8-42.3) | 12.1 ± 1.1 (7.8-16.7) | 16.4 ± 1.9 (12-23.4) | 10.1 ± 2.8 (3.1-30.4) | 13.7 ± 1.6 (7-20.7) |
| **DA** | 16.3 ± 2.3 (7.5-24.5) | 6.9 ± 0.9 (4-12.1) | 8.8 ± 1 (6.3-11.8) | 7.3 ± 1.4 (1.9-15.4) | 9.2 ± 1 (4.4-13.2) |
| **DC** | 144.9 ± 22.9 (62.2-259.8) | 59.9 ± 7.2 (35.6-102.9) | 79.8 ± 11.3 (58.5-120.3) | 60.6 ± 12.7 (17.2-147.4) | 75 ± 7.4 (40.2-102.3) |
| **FH** | 7.5 ± 1.1 (3.6-12) | 3.3 ± 0.5 (1.8-6.6) | 3.8 ± 0.5 (2.8-5.3) | 3.3 ± 0.6 (1-7.1) | 4.1 ± 0.4 (2-5.5) |
| **TH** | 68.3 ± 10.4 (30.9-97.9) | 27.5 ± 3.4 (12.3-45.8) | 36.7 ± 5.4 (22.8-53.1) | 26.8 ± 6.1 (7-70) | 40.3 ± 5 (19.1-63.6) |

**Supplementary Table 2. Normality tests of the study dataset.**

All 25 variables in our analogue sharks (*N* = 41) are tested, their results accurate to two decimal places. Raw data were Tukey transformed and subsequently displayed normal distribution (*P* > 0.05).

| Variable | W | P |
| --- | --- | --- |
| **TL** | 0.97 | 0.32 |
| **HL** | 0.96 | 0.11 |
| **SE** | 0.98 | 0.58 |
| **UJH** | 0.98 | 0.5 |
| **LJH** | 0.99 | 0.99 |
| **SP** | 0.95 | 0.09 |
| **GS** | 0.98 | 0.55 |
| **PecL** | 0.98 | 0.83 |
| **PecW** | 0.97 | 0.46 |
| **SD** | 0.95 | 0.08 |
| **DH** | 0.99 | 0.88 |
| **DW** | 0.98 | 0.75 |
| **DAP** | 0.98 | 0.75 |
| **DTA** | 0.98 | 0.57 |
| **DPA** | 0.97 | 0.46 |
| **DD** | 0.97 | 0.3 |
| **PP** | 0.97 | 0.26 |
| **PelL** | 0.99 | 0.91 |
| **PelW** | 0.98 | 0.64 |
| **BPA** | 0.97 | 0.33 |
| **PA** | 0.98 | 0.58 |
| **DA** | 0.97 | 0.4 |
| **DC** | 0.98 | 0.56 |
| **FH** | 0.98 | 0.8 |
| **TH** | 0.97 | 0.47 |

**Supplementary Table 3. Comparison of our model using all analogues *vs.* a model using the great white shark only (GWS).**

Both models are used to estimate body dimensions of a 16 m †*O. megalodon*. Measurements are accurate to two decimal places in cm. Mean proportions of ‘all species’ model is included in Table 1; statistical results of all linear regressions recorded are found in Supplementary Data 3.

| Variable | Model using all species | | | Model using the great white shark only | | | |
| --- | --- | --- | --- | --- | --- | --- | --- |
|  | 16m  Megalodon | R^2^ | P-value | GWS  mean proportion | 16m Megalodon | R^2^ | P-value |
| HL | 464.86 | 0.969 | 3.87e-31 | 0.29 | 462.76 | 0.974 | 5.36e-07 |
| SE | 79.69 | 0.719 | 1.6e-12 | 0.04 | 65.56 | 0.904 | 5.24e-05 |
| UJH | 118.66 | 0.956 | 3.12e-28 | 0.08 | 119.44 | 0.961 | 2.12e-06 |
| LJH | 56.95 | 0.717 | 1.81e-12 | 0.03 | 55.04 | 0.608 | 8.03e-03 |
| SP | 421.68 | 0.95 | 3.52e-27 | 0.26 | 413.22 | 0.945 | 7.51e-06 |
| GS | 141.19 | 0.917 | 6.23e-23 | 0.1 | 158.24 | 0.824 | 4.43e-04 |
| PecL | 308.42 | 0.825 | 1.53e-16 | 0.19 | 309.39 | 0.91 | 4.2e-05 |
| PecW | 164.79 | 0.91 | 4.11e-22 | 0.12 | 192.64 | 0.832 | 3.78e-04 |
| SD | 593.71 | 0.965 | 3.74e-30 | 0.38 | 613.52 | 0.98 | 1.67e-06 |
| DH | 162.48 | 0.908 | 5.32e-22 | 0.1 | 164.33 | 0.93 | 1.74e-05 |
| DW | 198.96 | 0.94 | 1.29e-25 | 0.13 | 201.16 | 0.967 | 1.15e-06 |
| DAP | 232.54 | 0.958 | 1.39e-28 | 0.14 | 227.03 | 0.964 | 1.62e-06 |
| DTA | 453.11 | 0.963 | 9.7e-30 | 0.27 | 433.88 | 0.984 | 1.01e-07 |
| DPA | 280.41 | 0.929 | 3.7e-24 | 0.16 | 259.13 | 0.873 | 1.38e-04 |
| DD | 373.43 | 0.965 | 3.48e-30 | 0.21 | 332.92 | 0.983 | 1.1e-07 |
| PP | 351.92 | 0.853 | 5.05e-18 | 0.21 | 338.57 | 0.72 | 2.36e-03 |
| PelL | 73.56 | 0.852 | 5.75e-18 | 0.06 | 88.59 | 0.667 | 4.42e-03 |
| PelW | 99.77 | 0.903 | 1.34e-21 | 0.07 | 112.39 | 0.916 | 3.3e-05 |
| BPA | 195.97 | 0.962 | 1.14e-29 | 0.11 | 181.17 | 0.948 | 5.91e-06 |
| PA | 150.35 | 0.879 | 1.16e-19 | 0.09 | 142.85 | 0.827 | 4.66e-04 |
| DA | 94.58 | 0.965 | 3.83e-30 | 0.06 | 89.53 | 0.953 | 4.24e-06 |
| DC | 808.43 | 0.98 | 6.71e-35 | 0.49 | 789.28 | 0.965 | 1.51e-06 |
| FH | 43.69 | 0.962 | 1.64e-29 | 0.03 | 41.85 | 0.957 | 3.14e-06 |
| TH | 385.36 | 0.809 | 8.51e-16 | 0.24 | 382.2 | 0.552 | 1.32e-02 |

**Supplementary Table 4. Model accuracy test.**

Body dimensions estimates for a ~7 m *C. carcharias* using our model (all species included) tested against a known specimen of the same size. Measurements are taken from the best available image of ‘Deep Blue’, a female *C. carcharias* thought to be the largest ever recorded (Supplementary Data 1,2). While her exact size has proven difficult to determine (ranged from 6.1-7 m TL), and is disagreed among biologists, ~7 m has been her largest estimated size. We therefore use this estimation here as the closest possible size to †*O. megalodon*. All measurements are accurate to two decimal places and are reported in cm. 22/24 variables were found to be accurate to our mean ± sd model predictions. Only DTA and DPA did not fall into these predictions; though it should be noted that Deep Blue was strongly suspected to be pregnant at the time the image was taken.

| Variable | Predicted | | Empirical | |
| --- | --- | --- | --- | --- |
|  | **Mean** | **Standard deviation (sd)** | **Measurement** | **Fits mean & sd (Y/N)** |
| HL | 203.38 | 18.25 | 212.98 | Y |
| SE | 34.87 | 11.18 | 42.05 | Y |
| UJH | 51.91 | 7.61 | 56.98 | Y |
| LJH | 24.91 | 9.53 | 33.91 | Y |
| SP | 184.48 | 20.19 | 181.78 | Y |
| GS | 61.77 | 9.91 | 66.71 | Y |
| PecL | 134.93 | 33.78 | 118.83 | Y |
| PecW | 72.1 | 13.69 | 63.34 | Y |
| SD | 259.75 | 21.94 | 274.03 | Y |
| DH | 71.09 | 15.55 | 66.47 | Y |
| DW | 87.05 | 13.13 | 81.4 | Y |
| DAP | 101.74 | 14.91 | 99.03 | Y |
| DTA | 198.23 | 24.39 | 223.84 | N |
| DPA | 122.68 | 17.61 | 147.87 | N |
| DD | 163.37 | 14.52 | 154.65 | Y |
| PP | 153.96 | 25.48 | 176.36 | Y |
| PelL | 32.18 | 7.97 | 26.34 | Y |
| PelW | 43.65 | 10.69 | 33.94 | Y |
| BPA | 85.74 | 8.53 | 79.19 | Y |
| PA | 65.78 | 12.26 | 61.05 | Y |
| DA | 41.38 | 4.21 | 40.7 | Y |
| DC | 353.69 | 20.58 | 359.5 | Y |
| FH | 19.11 | 2.13 | 17.64 | Y |
| TH | 168.6 | 30.53 | 198.06 | Y |


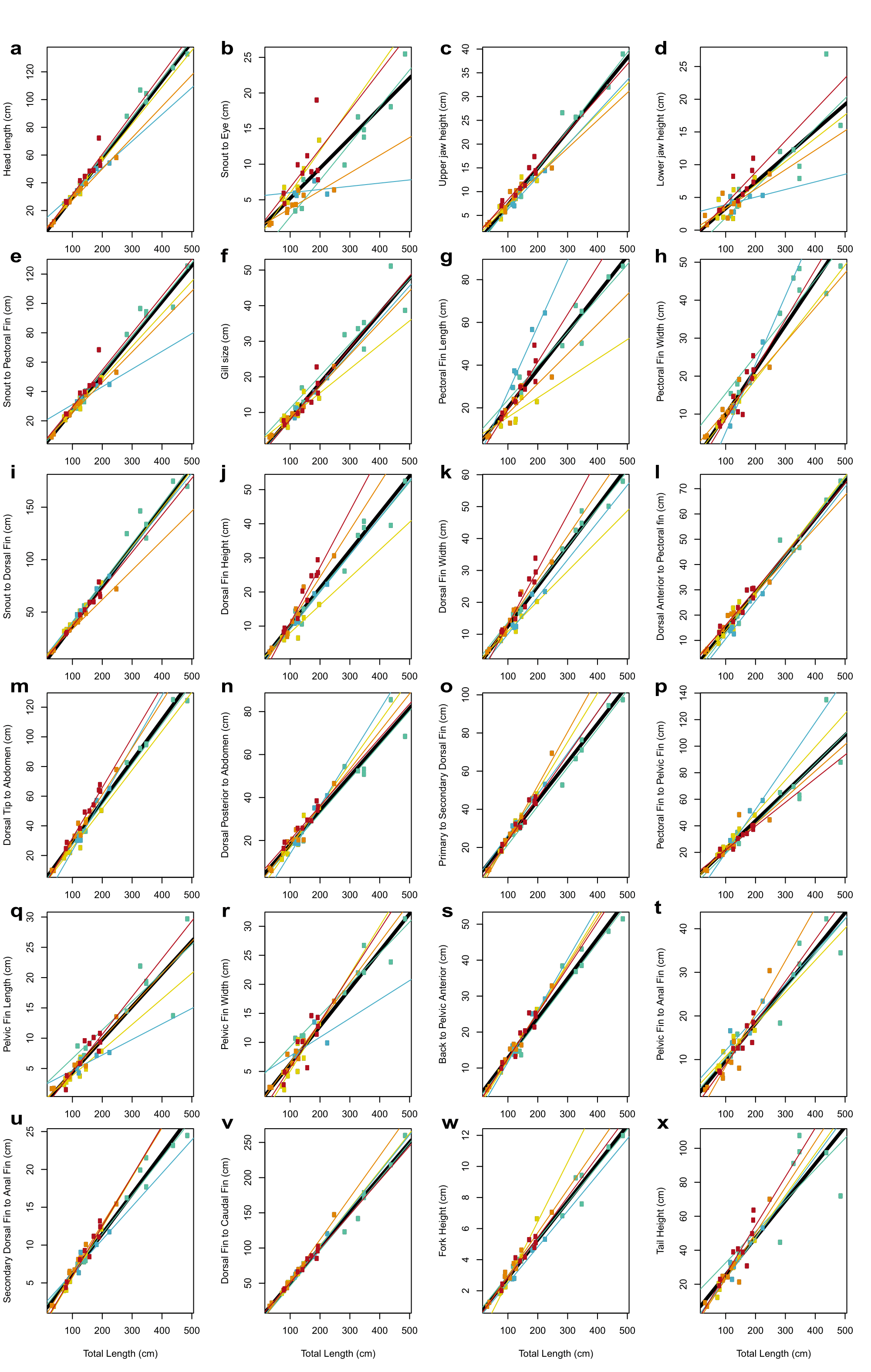


**Supplementary Fig. 1.** The linear relationship between TL (x axis) and all twenty-four anatomical variables (y axis). (**a**) Head length; (**b**) Snout-eye; (**c**) Upper jaw height; (**d**) Lower jaw height; (**e**) Snout-pectoral; (**f**) gill size; (**g**) Pectoral fin length; (**h**) Pectoral fin width; (**i**) Snout-dorsal; (**j**) Dorsal fin height; (**k**) Dorsal fin width; (**l**) Dorsal anterior-pectoral fin; (**m**) Dorsal tip-abdomen; (**n**) Dorsal posterior-abdomen; (**o**) Primary-secondary dorsal fin; (**p**) Pectoral-pelvic fin; (**q**) Pelvic fin length; (**r**) Pelvic fin width; (**s**) Dorsal side-pelvic anterior; (**t**) Pelvic-anal fin; (**u**) Secondary dorsal-anal fin; (**v**) Dorsal-caudal fin; (**w**) Fork height; (**x**) Tail height. A thick black line marks the linear regression considering all species. Each thinner, coloured line and data points represent individual species: green= *C. carcharias*; yellow = *I. oxyrinchus*; blue = *I. paucus*; orange = *L. ditropis*; red = *L. nasus*.


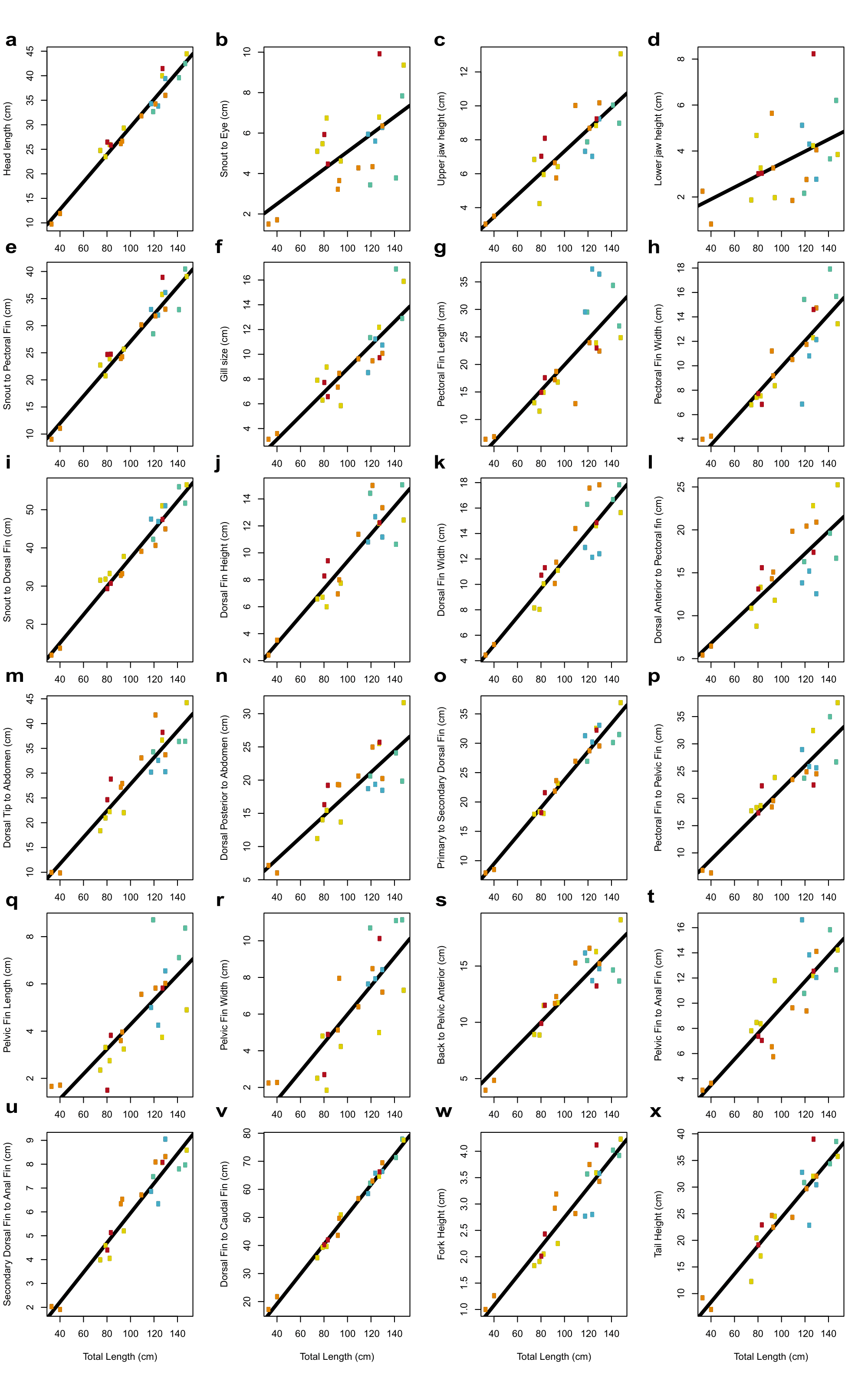


**Supplementary Fig. 2.** The linear relationship between TL (x axis) and all twenty-four anatomical variables (y axis) in juvenile analogue sharks. (**a**) Head length; (**b**) Snout-eye; (**c**) Upper jaw height; (**d**) Lower jaw height; (**e**) Snout-pectoral; (**f**) gill size; (**g**) Pectoral fin length; (**h**) Pectoral fin width; (**i**) Snout-dorsal; (**j**) Dorsal fin height; (**k**) Dorsal fin width; (**l**) Dorsal anterior-pectoral fin; (**m**) Dorsal tip-abdomen; (**n**) Dorsal posterior-abdomen; (**o**) Primary-secondary dorsal fin; (**p**) Pectoral-pelvic fin; (**q**) Pelvic fin length; (**r**) Pelvic fin width; (**s**) Dorsal side-pelvic anterior; (**t**) Pelvic-anal fin; (**u**) Secondary dorsal-anal fin; (**v**) Dorsal-caudal fin; (**w**) Fork height; (**x**) Tail height. A thick black line marks the linear regression considering all species. Coloured points represent individual species: green= *C. carcharias*; yellow = *I. oxyrinchus*; blue = *I. paucus*; orange = *L. ditropis*; red = *L. nasus*.


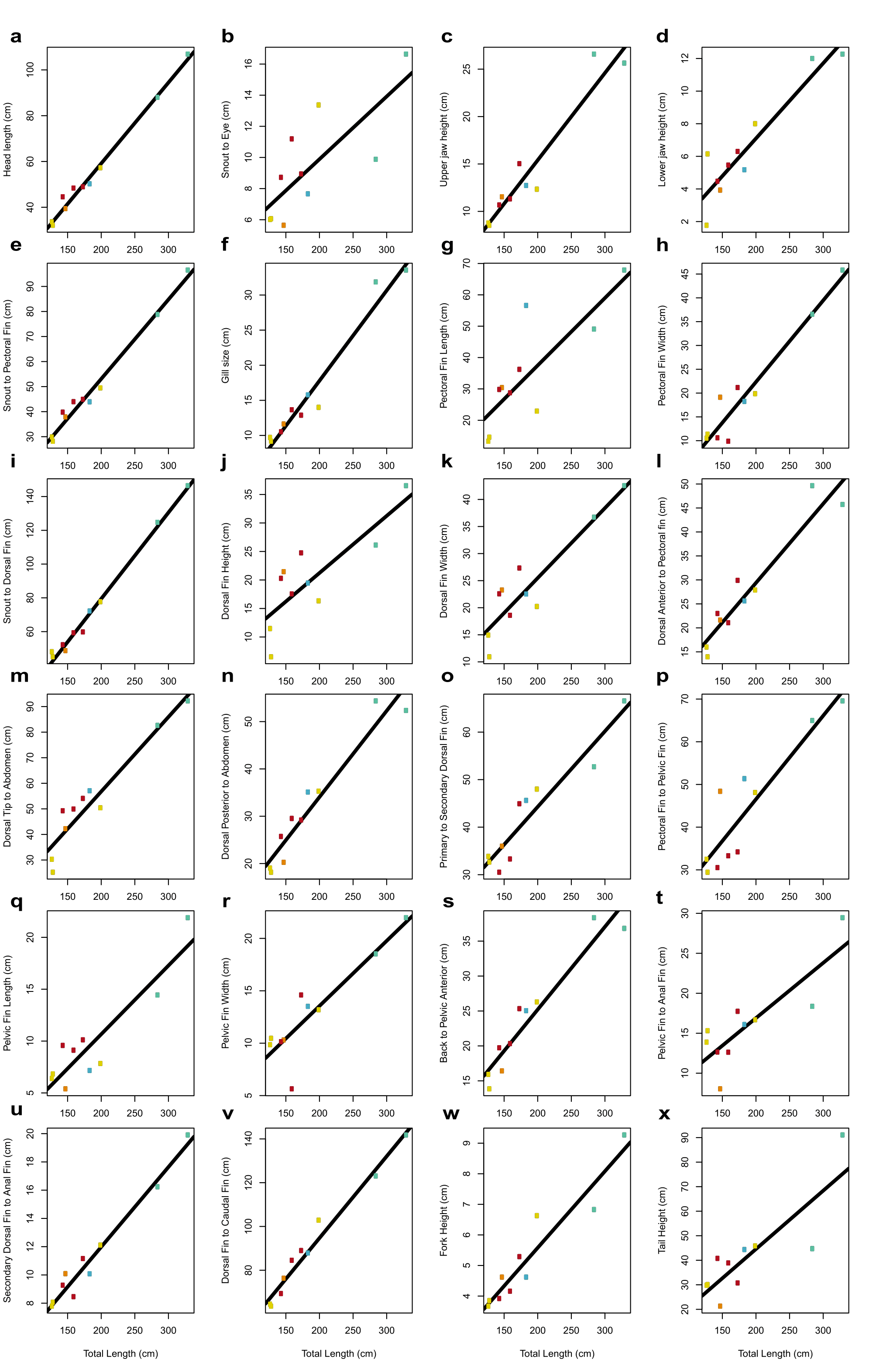


**Supplementary Fig. 3.** The linear relationship between TL (x axis) and all twenty-four measured variables (y axis) in subadult analogue sharks. (**a**) Head length; (**b**) Snout-eye; (**c**) Upper jaw height; (**d**) Lower jaw height; (**e**) Snout-pectoral; (**f**) gill size; (**g**) Pectoral fin length; (**h**) Pectoral fin width; (**i**) Snout-dorsal; (**j**) Dorsal fin height; (**k**) Dorsal fin width; (**l**) Dorsal anterior-pectoral fin; (**m**) Dorsal tip-abdomen; (**n**) Dorsal posterior-abdomen; (**o**) Primary-secondary dorsal fin; (**p**) Pectoral-pelvic fin; (**q**) Pelvic fin length; (**r**) Pelvic fin width; (**s**) Dorsal side-pelvic anterior; (**t**) Pelvic-anal fin; (**u**) Secondary dorsal-anal fin; (**v**) Dorsal-caudal fin; (**w**) Fork height; (**x**) Tail height. A thick black line marks the linear regression considering all species. Coloured points represent individual species: green= *C. carcharias*; yellow = *I. oxyrinchus*; blue = *I. paucus*; orange = *L. ditropis*; red = *L. nasus*.


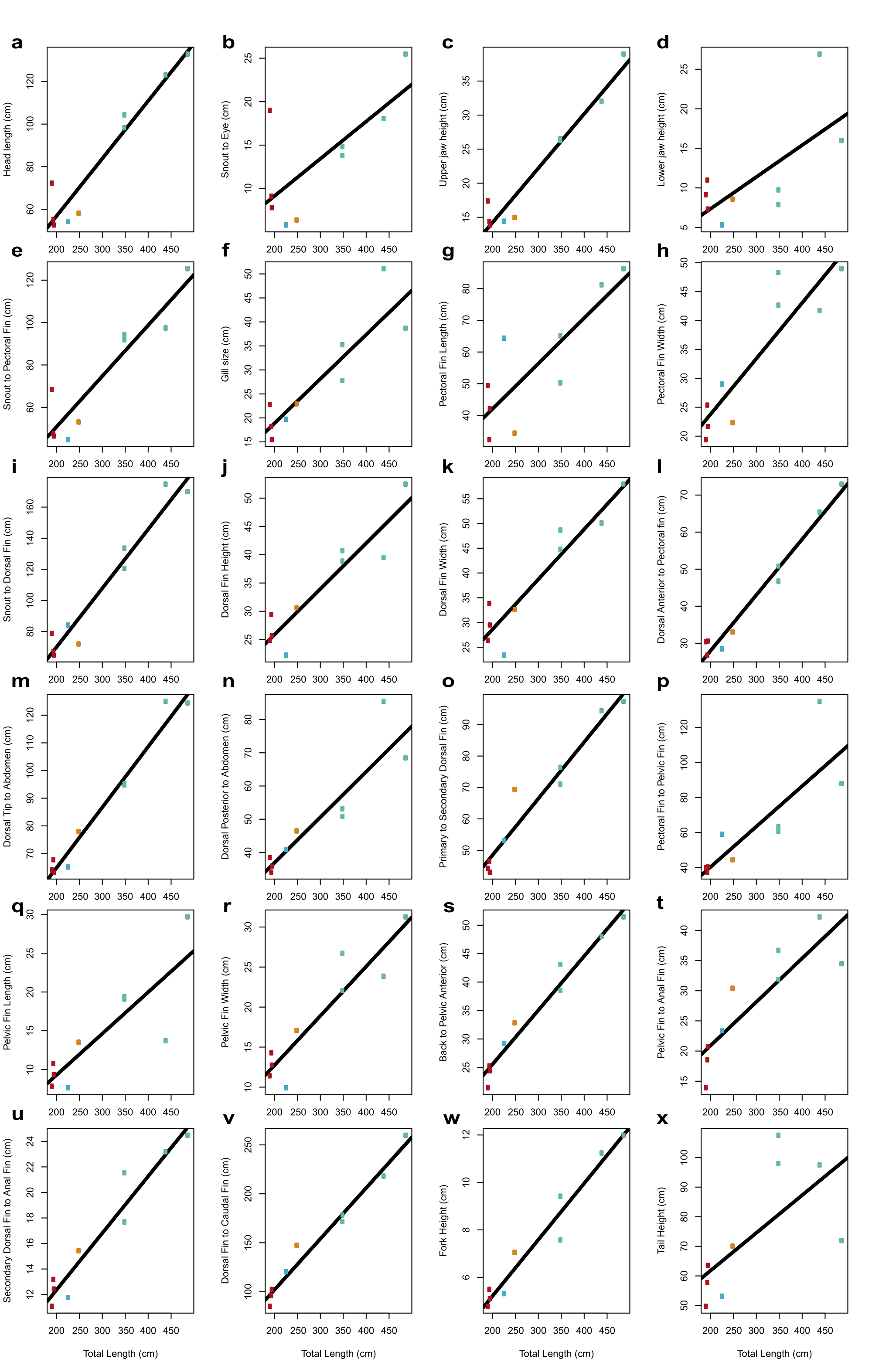


**Supplementary Fig. 4.** The linear regression results of all twenty-four measured variables (y axis) in our adult sharks against TL (x axis). (**a**) Head length; (**b**) Snout-eye; (**c**) Upper jaw height; (**d**) Lower jaw height; (**e**) Snout-pectoral; (**f**) gill size; (**g**) Pectoral fin length; (**h**) Pectoral fin width; (**i**) Snout-dorsal; (**j**) Dorsal fin height; (**k**) Dorsal fin width; (**l**) Dorsal anterior-pectoral fin; (**m**) Dorsal tip-abdomen; (**n**) Dorsal posterior-abdomen; (**o**) Primary-secondary dorsal fin; (**p**) Pectoral-pelvic fin; (**q**) Pelvic fin length; (**r**) Pelvic fin width; (**s**) Dorsal side-pelvic anterior; (**t**) Pelvic-anal fin; (**u**) Secondary dorsal-anal fin; (**v**) Dorsal-caudal fin; (**w**) Fork height; (**x**) Tail height. A thick black line marks the linear regression considering all species. Coloured points represent individual species: green= *C. carcharias*; yellow = *I. oxyrinchus*; blue = *I. paucus*; orange = *L. ditropis*; red = *L. nasus*.


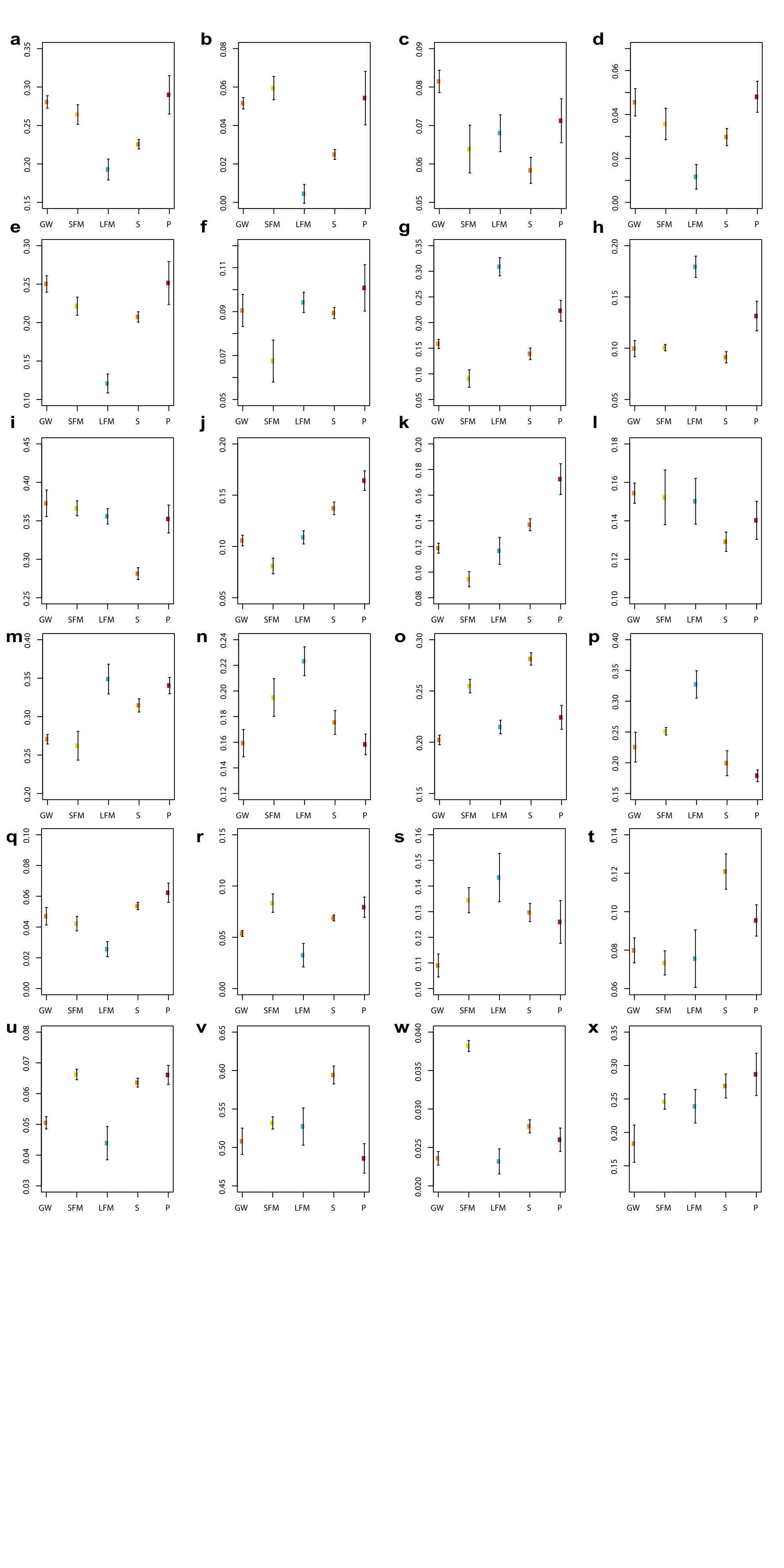


**Supplementary Fig. 5.** Slopes of the linear regressions of all twenty-four variables against TL, and standard errors. (**a**) Head length; (**b**) Snout-eye; (**c**) Upper jaw height; (**d**) Lower jaw height; (**e**) Snout-pectoral; (**f**) gill size; (**g**) Pectoral fin length; (**h**) Pectoral fin width; (**i**) Snout-dorsal; (**j**) Dorsal fin height; (**k**) Dorsal fin width; (**l**) Dorsal anterior-pectoral fin; (**m**) Dorsal tip-abdomen; (**n**) Dorsal posterior-abdomen; (**o**) Primary-secondary dorsal fin; (**p**) Pectoral-pelvic fin; (**q**) Pelvic fin length; (**r**) Pelvic fin width; (**s**) Dorsal side-pelvic anterior; (**t**) Pelvic-anal fin; (**u**) Secondary dorsal-anal fin; (**v**) Dorsal-caudal fin; (**w**) Fork height; (**x**) Tail height. Colours represent each individual species, abbreviated according to their common name, as in Supplementary Fig. 1-4.


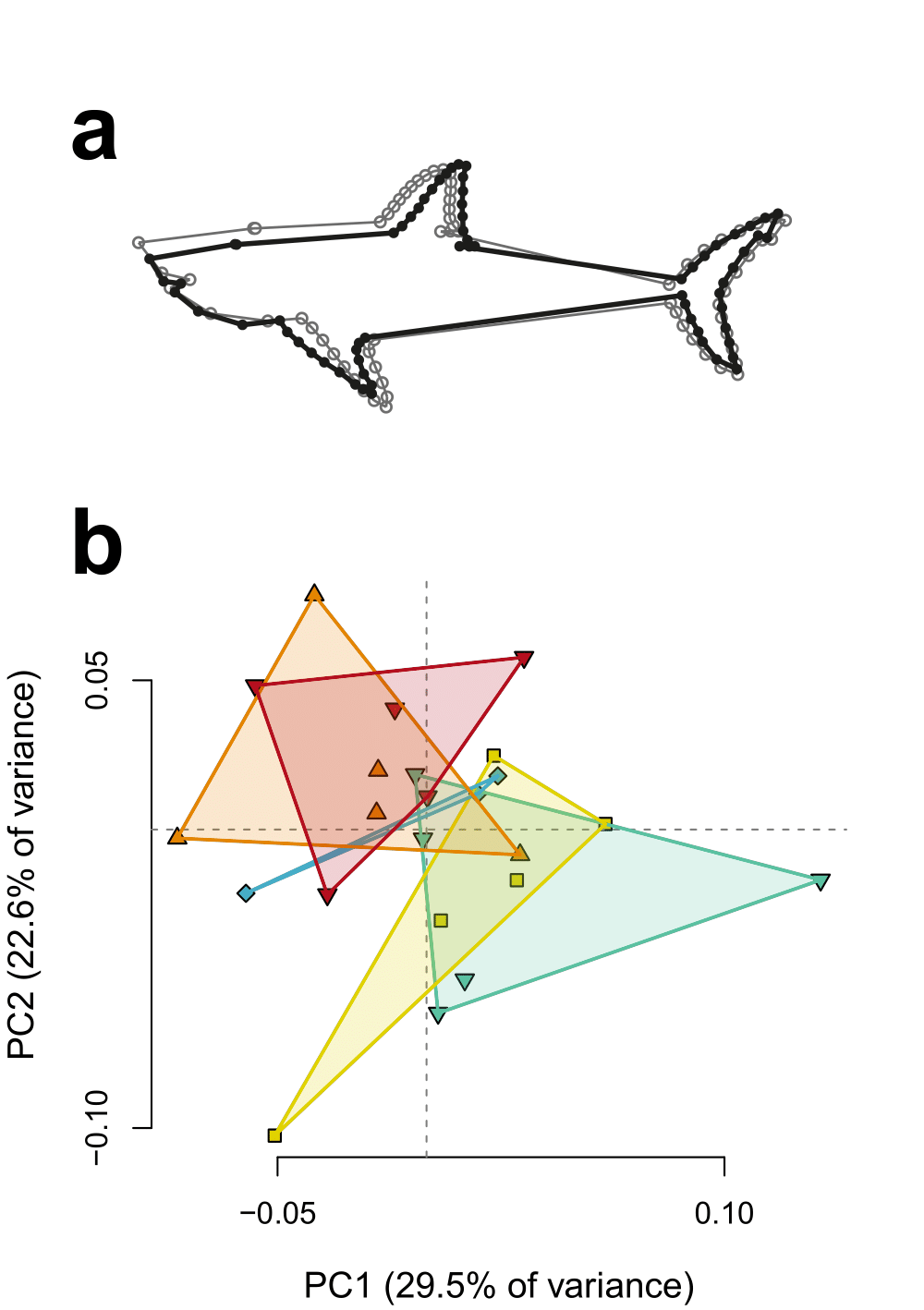


**Supplementary Fig. 6.** Generalised regression shape changes and PCA for total body analyses. (**a**) Generalised regression shape changes of Procrustes coordinates observed within the bodies of our analogues sharks when all landmarks are analysed within a single configuration. Light and dark grey configurations represent the morphological change occurring from the average shape towards higher scores, considering in all cases a magnitude of the shape change equal to 0.1. The trends observed in Fig. 1 shape changes are once again seen when all four body regions (head; pectoral, dorsal and caudal fin) are analysed as a single landmark configuration. Shape changes were visualised in MorphoJ^6^ (**b**) PCA of Procrustes coordinates. Morphospace overlap is again apparent even when all landmarks are used. Individual colours represent each species: green = *Carcharodon carcharias*; yellow = *Isurus oxyrinchus*; blue = *Isurus paucus*; orange = *Lamna ditropis*; red = *Lamna nasus*. PCA plot was created in R^7^.

**
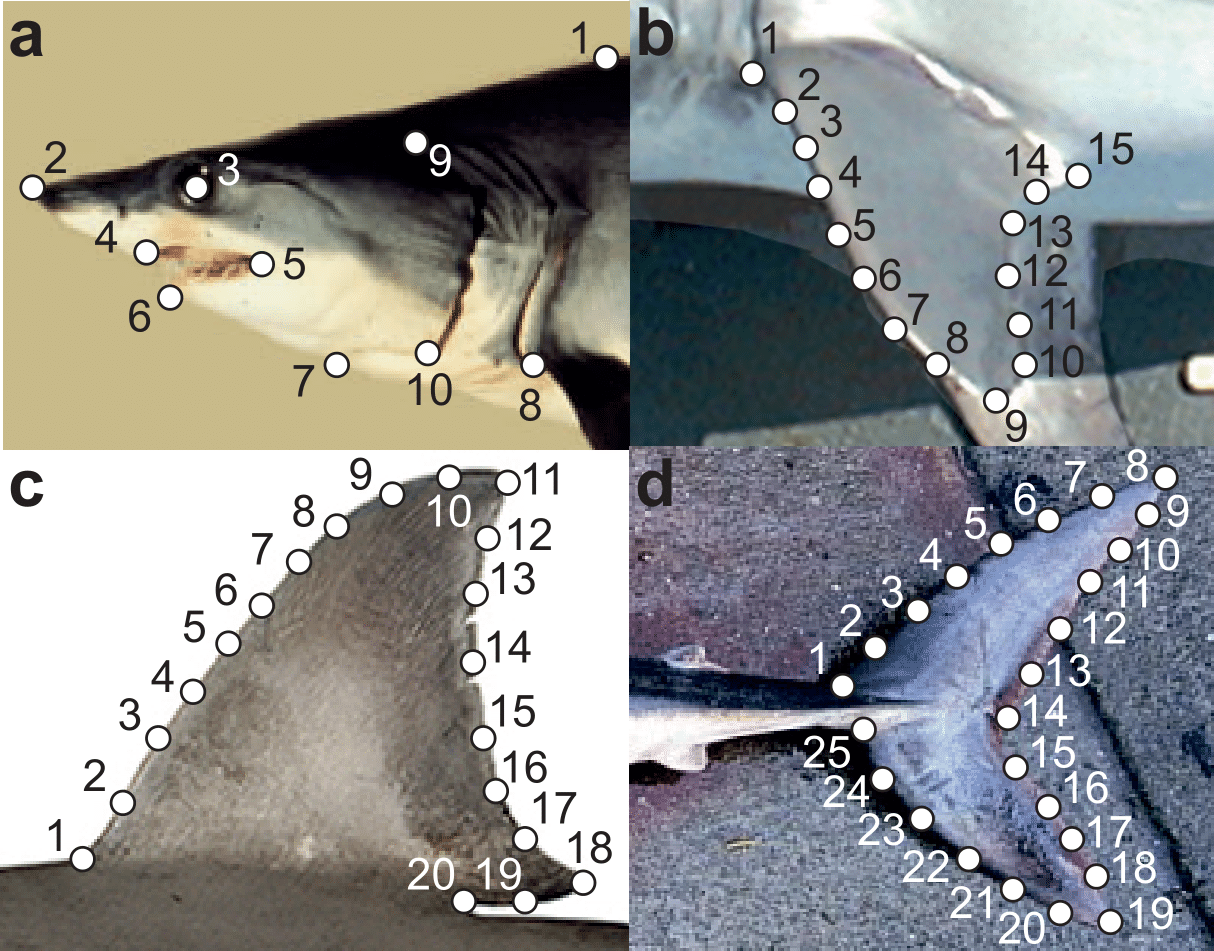
**

**Supplementary Fig. 7.** Landmark configurations used in morphometric analyses to digitise the shape of the (**a**) head; (**b**) pectoral fin; (**c**) dorsal fin; and (**d**) caudal fin. All 41 images collected were used in our analyses. Here we show examples of four individuals [in brackets] and species: (**a**) [ShortfinMako5] *Isurus oxyrinchus*; (**b**) [Porbeagle7] *Lamna nasus*; (**c**) [GreatWhite5] *Carcharodon carcharias*; and (**d**) [Porbeagle6] *Lamna nasus*. These particular individuals can be found in Supplementary Data 2 and sources of these images are listed in Supplementary Data 1.

**c**

**b**

**a**

**d**

**f**

**e**

**h**

**g**

**Supplementary Fig. 8.** GPA (**a**,**c**,**e**,**g**) and RFTRA (**b**,**d**,**f**,**h**) superimpositions for the head shape (**a**,**b**), pectoral fin (**c**,**d**), dorsal fin (**e**,**f**) and caudal fin (**g**,**h**) of the analogue species used in the study. Points denote landmarks and crosses denote landmark centroids. Axes represent relative positions on landmarks after superimposition.

**Supplementary references**

1. Compagno, L. J. V. Sharks of the world: an annotated and illustrated catalogue of shark species known to date. FAO Species Catalogue for Fishery Purposes **2**, 269 (Food & Agriculture Org., 2001).
2. Gottfried, M. D., Compagno, L. J. V. & Bowman, S. C. Size and skeletal anatomy of the giant “megatooth” shark *Carcharodon megalodon*. In *Great White Sharks: the biology of Carcharodon carcharias* (eds. Klimley, A. P. & Ainley, D. G.), Ch. 7 (Academic Press, San Diego, 1996).
3. Pratt Jr, H. L. & Casey, J. G. Age and growth of the shortfin mako, *Isurus oxyrinchus*, using four methods. *Can. J. Fish. Aquat. Sci.* **40**, 1944–1957 (1983).
4. Goldman, K. J. & Musick, J. A. Growth and maturity of salmon sharks (*Lamna ditropis*) in the eastern and western North Pacific, and comments on back-calculation methods. *Fish. Bull.* **104**, 278–292 (2006).
5. Natanson, L. J., Mello, J. J. & Campana, S. E. Validated age and growth of the porbeagle shark (*Lamna nasus*) in the western North Atlantic Ocean. *Fish. Bull.* **100**, 266–278 (2002).
6. Klingenberg, C. P. MorphoJ: an integrated software package for geometric morphometrics. *Mol. Ecol. Resour.* **11**, 353–357 (2010).
7. R Development Core Team. *R: A language and environment for statistical computing*, 3.4.2 Edition. R Foundation for Statistical Computing, Vienna (2017).
